# Amikacin resistance due to the *aphA6* gene in multi-antibiotic resistant *Acinetobacter baumannii* isolates belonging to global clone 1 from Iran

**DOI:** 10.1186/s12866-019-1592-6

**Published:** 2019-09-18

**Authors:** Parisa Aris, Mohammad Ali Boroumand, Masoumeh Douraghi

**Affiliations:** 1 Division of Microbiology, Department of Pathobiology, School of Public Health, Tehran University of Medical Sciences, PO Box: 14155-6446, Tehran, Iran; 2 Department of Pathology, Tehran Heart Center, Tehran University of Medical Sciences, Tehran, Iran; 3 Food Microbiology Research Center, Tehran University of Medical Sciences, Tehran, Iran

**Keywords:** *Acinetobacter baumannii*, Amikacin resistance, *aphA6*, Iran

## Abstract

**Background:**

Tn*aphA6*-carrying *repAci6* plasmids have been detected in *Acinetobacter baumannii* isolates belonging to global clones, GC1 and GC2, worldwide. Here, we examined whether RepAci6 plasmids family play a role in the dissemination of the *aphA6* in GC1 *A. baumannii* isolates from Iran.

**Results:**

We found that 22 isolates carried the *repAci6* gene, suggesting that they contain a RepAci6 plasmid family. Using the primers linking the *aphA6* gene to the backbone of *repAci6* plasmid**,** it was revealed that 16 isolates from different hospitals harbored Tn*aphA6* on a *repAci6* plasmid.

**Conclusions:**

This study provides evidence for the dissemination of Tn*aphA6* on the plasmids encoding RepAci6 in Iranian *A. baumannii* isolates. Furthermore, it seems that Tn*aphA6* might be acquired by distinct plasmids separately as it was found to be located on the variants of *repAci6* plasmids.

## Background

Multi-antibiotic resistant (MAR) *Acinetobacter baumannii* infections are mostly associated with strains belonging to global clone 1 (GC1) and GC2 [1]. Carbapenems have remained the antibiotics of choice for the treatment of infections caused by MAR *A. baumannii* strains [2]. However, carbapenem resistance has also become widespread in both GC1 and GC2 *A. baumannii* strains [2]. Aminoglycosides are a potent alternative for the treatment of carbapenem-resistant *A. baumannii* infections [3]. Resistance to aminoglycosides in *A. baumannii* primarily arises through the genes encoding aminoglycoside-modifying enzymes [4]. Of the genes conferring resistance to aminoglycosides, the *aphA6* gene encodes aminoglycoside 3′-phosphotransferase. It was first found on a 63-kb plasmid in a kanamycin-amikacin resistant *A. baumannii* in 1988. This plasmid, pIP1841, was shown to be transferable to other *Acinetobacter* species [5]. In *Acinetobacter* species, the *aphA6* gene is commonly found to be flanked by copies of ISAba125 in a composite transposon namely Tn*aphA6*. It is demonstrated that ISAba125 plays a role in overexpression of the *aphA6* and high-level resistance to amikacin, kanamycin, and neomycin [6, 7]. Tn*aphA6* was first detected in the Australian isolates belonging to global clone 2 (GC2), then in a member of global clone 1 (GC1) from Australia [8, 9], and lately in the isolates containing pCMCVT*Ab*2-type plasmids from the United States [10]. Tn*aphA6* was also found on pACICU2 in the same location as pAb-G7–2 [11]. These plasmids belong to the most common plasmid family which encodes RepAci6 [9–12]. The plasmids in *A. baumannii* are categorized into 19 distinct groups on the basis of the nucleotide homology of their respective replicase genes [12]. There are a wide variety of variants of *repAci6* plasmids and some have been shown to be conjugative [6, 8, 11]. The ubiquitous occurrence of *repAci6* plasmid was demonstrated in MAR *A. baumannii* isolates from 17 European countries [13]. The majority of *A. baumannii* isolates from European countries (96.8%) and half of the isolates from Asian countries (51.5 and 46.9% in Chinese and Taiwanese isolates, respectively) were found to carry *repAci6* plasmid [13–15]. The RepAci6 plasmids are important because some of them are associated with the carriage of the resistance genes, including the *aphA6,* and contribute to the dissemination of the resistance to amikacin [6, 8, 11]. Amikacin resistance due to the Tn*aphA6* has been shown in the isolates from some countries such as Australia, Italy, and United States [8–11].

In the recent years, a remarkable increase in the resistance to amikacin has been reported – from 58.4% in 2001–2007 to 95% in 2012–2013 – in Iran [16], where the *aphA6* gene is widespread ranging from 38.8 to 90% [17–19]. In this study, we examined whether plasmids encoding RepAci6 play a role in the dissemination of the *aphA6* gene in GC1 *A. baumannii* isolates from Iran.

## Results

### Phenotype observed

With respect to phenotypic resistance to amikacin, all the 55 isolates were non-susceptible to amikacin (52 resistant and 3 intermediate susceptible). The antibiotic resistance profile of each strain had been provided through the supplementary data file (see www.karger.com/doi/10.1159/000448785 for all online suppl. material) [20]. Furthermore, the properties of *A. baumannii* isolates tested are shown in Additional file 1: Table S1.

### *aphA6* screening

In total, the *aphA6* gene was detected in 54 of the 55 isolates tested. The 52 amikacin-resistant isolates and all but one of the intermediate susceptible isolates harbored the *aphA6* gene.

### Detecting *aphA6* in Tn*aphA6*

The *aphA6* gene is located in Tn*aphA6* of 52 isolates out of 54 *aphA6*-harboring isolates as the RH573-aphA6R and aphA6F-RH574 PCRs join the *aphA6* gene to ISAba125, indicating that the *aphA6* is contained within the Tn*aphA6*. The remaining 2 isolates, ABS064 and ABS258, (Additional file 1: Table S1) harbored the *aphA6* gene, which was not bound by ISAba125, as the linkage PCRs did not generate amplicons.

### Detecting Tn*aphA6* in Aci6 plasmid backbones

The screening of the *aci6* replication initiation gene revealed that 22 isolates carried the *repAci6* gene. Only 16 isolates were found to harbor Tn*aphA6* on the RepAci6 plasmid. In terms of the presence or absence of PCR amplicons for the different segments of the *repAci6* plasmid, these isolates fell into the following groups: i) group 1 included 14 out of 16 isolates from five different hospitals were found to harbor Tn*aphA6* on a *repAci6* plasmid (Table 1). The RH1501-aphA6R and aphA6F-RH1502 linkage PCRs produced amplicons consistent with the predicted amplicon sizes of 1930 bp and 2540 bp, respectively. The backbone of *repAci6* plasmids is comprised of three segments separated by a repeated sequence. Repeat 1 is 225 bp, while repeats 2 and 3 are 422 or 423 bp [9]. Here, we examines if these sequences are present. The PCRs for repeated sequences 1, 2, and 3 produced the expected amplicons, suggesting the presence of a *repAci6* plasmid. The sequencing of Tn*aphA6* besides the repeated sequences 1, 2, and 3 showed that it shares 100% nucleotide identity to some plasmids such as pAb-G7–2, pCMCVTAb2-Ab4, pC13–2, and p1AB5075, which belong to the RepAci6 plasmid family. The complete sequence of Tn*aphA6* and 4130 bp its flanking sequences were determined from *A. baumannii* strain ABS201 and deposited in the GenBank under the accession number MH500097; ii) the isolate ABI032 (group 2) had a RepAci6 plasmid and generated amplicons consistent with the expected sizes for all the PCRs, except for the repeated sequence 3. The amplicon size of repeated sequence 3 in this isolate was larger than the expected amplicon size of 1575 bp and was approximately 1800 bp. The sequencing of this larger amplicon showed that it shares 99% nucleotide identity to that of p1AB5075 belongs to the RepAci6 plasmid family [21]; iii) the isolate ABS224 (group 3) also had a RepAci6 plasmid, but did not produce an amplicon for Tn*aphA6*_R, yielded a larger amplicon for repeated sequence 3 and generated all the other amplicons with the expected sizes in PCRs indicated in Table 2. The sequencing of the repeated sequence 3 in addition to repeated sequences 1 and 2 showed that it shares 99% nucleotide identity to that of pUSA15 belongs to the RepAci6 plasmid family [23]; iv) group 4 included the remaining 6 out of 22 *repAci6-*carrying isolates, did not produce the amplicons for PCRs linking the *aphA6* to the backbone of *repAci6* plasmids, Tn*aphA6*_L and Tn*aphA6*_R, and generated a larger amplicon for repeated sequence 3 (Table 1). The 33 isolates did not produce amplicon for the *aci6* replicon gene. Thirty out of 33 isolates harbored Tn*aphA6*, but there was no evidence to locate Tn*aphA6* on plasmids encoding RepAci6 in these isolates. Most of these isolates (29 out of 30) were from the hospital H5. Of the remaining 3 isolates, 2 carried the *aphA6* gene which was not bound by ISAba125 and 1 isolate lacked the *aphA6* gene.

**Table 1:** Properties of 22 isolates containing *repAci6* plasmids

Isolate	Number of isolates	Year	Hospital	*repAci6*	*aphA6*	Tn*aphA6*	Tn*aphA6*_L	Tn*aphA6*_R	Repeated sequence 1	Repeated sequence 2	Repeated sequence 3
Group 1				**+**	**+**	**+**	**+**	**+**	**+**	**+**	**+**
ABS103, ABS178,	14	2011–2013	H1, H2, H3,								
ABS180, ABS226,			H4, H5								
ABS290, ABS249,											
ABS288, ABH008, ABS104, ABS200,											
ABS201, ABM015,											
ABI031, ABS094											
Group 2				**+**	**+**	**+**	**+**	**+**	**+**	**+**	**+** ^**a**^
ABI032	1	2012	H3								
Group 3				**+**	**+**	**+**	**+**	**–**	**+**	**+**	**+** ^**a**^
ABS224	1	2013	H5								
Group 4				**+**	**+**	**+**	**–**	**–**	**+**	**+**	**+** ^**a**^
ABS101, ABS115,	6	2013	H5								
ABS138, ABS267,											
ABS078, ABS084											

^**a**^The amplicon is larger than the expected size

**Table 2:** The primers used in this study

Target	Primer	Sequence (5′-3′)	Annealing temperature (°C)	Amplicon length (bp)	Reference
*aphA6*	aphA6F	CATTTGCGGGGTTTTTAATG	59	837	This study
	aphA6R	TTAGATAATGCTTGGAATCA			
ISAba125*-aphA6*	RH573	AAGAAGGCTTTTCAGCCAGA	58	1427	[8]
	aphA6R	GGACAATCAATAATAGCAAT			[22]
*aphA6-*ISAba125	aphA6F	ATACAGAGACCACCATACAGT	58	1745	[22]
	RH574	CAAACATGAGGTGCGACAGT			[8]
*repAci6*	gr6FW	AGCAAGTACGTGGGACTAAT	59	662	[12]
	gr6RV	AAGCAATGAAACAGGCTAAT			[12]
Tn*aphA6*_L	RH1501	CTTGAGGAAGGGATGGTTGA	59	1930	[9]
	aphA6R	GGACAATCAATAATAGCAAT			[22]
Tn*aphA6*_R	aphA6F	ATACAGAGACCACCATACAGT	59	2540	[22]
	RH1502	TTGCTTTAATCGGTGGTTCC			[9]
Repeated sequence 1	RH1398	TTTGACGTTGCTCTTGTTGC	58	991	[9]
	RH1399	TTCTCCCAAGTGGTCAGGTC			[9]
Repeated sequence 2	RH1394	TGGTTGGCAGAACAAGATGA	58	1372	[9]
	RH1395	TCAAACGATGCAATGGAAGA			[9]
Repeated sequence 3	RH1503	GAAGATCCAGAAGCGGGATA	58	1575	[9]
	RH1397	CCATGTTCTTTTCCACATGC			[9]

## Discussion

To date, Tn*aphA6*-carrying plasmids belonging to *repAci6* plasmid family have appeared to be mainly restricted to the Australian isolates and have only occasionally been found in the limited number of isolates from Italy [11], Romania [24], and the United States [10]. The *aphA6* has been detected not only in *A. baumannii* but also on a plasmid of *Escherichia coli* and *Klebsiella pneumoniae* isolates from Republic of the Union of Myanmar, Australia, and Italy [25–27]. In addition, this gene was found on a plasmid of *Providencia rettgeri* isolate from Canada in 2013 [28].

In this study, all isolates harbored *aphA6* except one with intermediate susceptibility to amikacin which lacked the *aphA6* gene, suggesting that other mechanisms may contribute to the resistance of this isolate to amikacin. Moreover, it was found that the *aphA6* in majority of the isolates is located in Tn*aphA6*. This finding is consistent with the previous studies reporting that the *aphA6* gene is frequently located in Tn*aphA6* [8–10].

It was observed that nearly half of the isolates carried *repAci6* gene, suggesting that they contain the RepAci6 plasmid family. These isolates fell into the four groups: in group 1, which accounted for three-quarters of the isolates, the positive results of PCRs spanning the *aphA6* gene to the backbone of plasmids encoding RepAci6 on the right and left indicate that the Tn*aphA6* is likely to be present in a *repAci6* plasmid family. The only isolate of group 2, had a RepAci6 plasmid and generated amplicons consistent with the expected sizes for all the PCRs, except for the repeated sequence 3. The amplicon size of repeated sequence 3 in this isolate was larger than the expected amplicon size and may imply that Tn*aphA6* is located in a different position on the *repAci6* plasmid from the isolates that had a usual size for repeated sequence 3. This finding indicates that Tn*aphA6* is probably present on a variant of *repAci6* plasmid family in this isolate (ABI032). With regard to the isolate in group 3, our finding probably implies the carriage of Tn*aphA6* in a different position on a variant of *repAci6* plasmid family. The Tn*aphA6* in the last group of the isolates appears to be probably located on the variants of RepAci6 plasmids or on the other plasmid families or somewhere on the chromosome in these isolates.

Most of the remaining isolates harbored Tn*aphA6*, but there was no evidence to locate Tn*aphA6* on plasmids encoding RepAci6 in these isolates; therefore, Tn*aphA6* might be located on the other plasmid families or somewhere on the chromosome.

The limitation of the current study is that all isolates were collected from the hospitals which were located in the same city. Collecting isolates from geographically distant locations in Iran will provide more comprehensive data and conducting such a study can be done in future.

## Conclusions

Using the PCR strategy previously developed [9], this study provides evidence for the dissemination of Tn*aphA6* on the *repAci6* plasmids family in amikacin-resistant isolates in Tehran, Iran (the second largest country in the Middle East). It appears that these plasmids are widespread in GC1 and 2 strains in the other parts of the world as we detected it in GC1 isolates from different hospitals within Tehran. In addition to the dissemination of Tn*aphA6* through this plasmid family and the emergence of resistance to aminoglycosides, Tn*aphA6* might be acquired by each distinct plasmid separately. Further studies are required in order to locate Tn*aphA6* in other isolates.

## Methods

### Bacterial isolates and antimicrobial susceptibility testing

A set of 55 multi-antibiotic resistant *A. baumannii* isolates, which had been previously assigned to GC1 were included in this study [20]. They were collected from 5 hospitals (H1–H5) between 2011 and 2013 in Tehran, Iran. For each strain, at least two overnight cultures in trypticase soy broth (TSB) with 15% glycerol from the first sub-culture of isolates were stored and then kept at − 70 °C until further experiments. The identity of *A. baumannii* isolates was confirmed by PCR assays targeting *gyrB* [29] and/or *bla*_OXA51-like_ [30] genes. In addition to the antibiotic resistance profile determined previously [20], susceptibility testing to amikacin (30 μg), kanamycin (30 μg), and neomycin (30 μg) was performed by disk diffusion method (Mast Diagnostics Ltd., Bootle, Merseyside, UK), and the results were interpreted according to the guidelines of the Clinical and Laboratory Standards Institute (CLSI) and the Calibrated Dichotomous Sensitivity (CDS) [31] (http://web.med.unsw.edu/cdstest). For the strains which were non-susceptible to amikacin by standard disk diffusion method, the minimum inhibitory concentration (MIC) of amikacin (Sigma Aldrich, St Louis, Missouri, USA) was determined. The MIC values were categorized as susceptible (S), intermediate (I), or resistant (R) according to the CLSI guidelines [31]. The protocol followed for disk diffusion and microbroth dilution method is available in the supplementary file.

*A. baumannii* strain NIPH 10 (provided by Alexander Nemec) was used as a positive control in PCR reaction when screening for the *aphA6* gene [3]. As the complete sequence of Tn*aphA6* in *A. baumannii* strain ABS201 was deposited in the GenBank (accession number MH500097) during the study period, it was also used as positive control. For negative control in PCR reaction, the DNA template was replaced with DNA of a strain lacking *aphA6* gene and sterile distilled water.

### Determining the context of *aphA6*

PCR was used to examine the presence of the *aphA6* gene and also to determine if it is located in Tn*aphA6*. PCR was also used to examine whether Tn*aphA6* is present in a context similar to the backbone of plasmids encoding RepAci6. Unique sequences on either side of the repeated sequence 1, 2 and 3 (often found in *repAci6* plasmids) were joined using previously designed PCRs [9], in cases that Tn*aphA6* was found in a context similar to the backbone of plasmids that carry the *repAci6* gene. All primers and their target segments for PCR amplifications are listed in Table 2. The sequencing of the representative PCR amplicons was performed on an Applied Biosystems using the ABI PRISM 3730xl DNA Analyzer (Macrogen Inc., South Korea), and the DNA sequences were analyzed using the online BLAST (http://blast.ncbi.nlm.nih.gov/BLAST.cgi).

## Supplementary information

**Additional file 1: Table S1.** Properties of *Acinetobacter baumannii* isolates studied.

## Data Availability

The datasets used and/or analyzed during the current study are available from the corresponding author on reasonable request. All data generated or analyzed during this study are included in this published article and its supplementary information file.

## Abbreviations

CDS: Calibrated Dichotomous Sensitivity
CLSI: Clinical and Laboratory Standards Institute
GC1: Global clone 1
GC2: Global clone 2
MAR: Multi-antibiotic resistant
MIC: Minimum inhibitory concentration
