# Supplementary material for: Amikacin resistance due to the aphA6 gene in multi-antibiotic resistant Acinetobacter baumannii isolates belonging to global clone 1 from Iran
Source: BMC Microbiol. 2019 Sep 18;19:221. doi: 10.1186/s12866-019-1592-6 (PMC6751817; doi:10.1186/s12866-019-1592-6)
Supplement: Supplementary file 1 — Additional file 1: Table S1. Properties of Acinetobacter baumannii isolates studied. [file 12866_2019_1592_MOESM1_ESM.docx]

Table S1. Properties of *Acinetobacter baumannii* isolates studied

| Isolate | Year | Hospital | Source | *aphA6* | ISAba125-*aphA6* | *aphA6*-ISAba125 | Tn*aphA6*_L | Tn*aphA6*_R | Repeated sequence 1 | Repeated sequence 2 | Repeated sequence 3 | *repAci6* | Aminoglycoside resistance phenotype | | |
| --- | --- | --- | --- | --- | --- | --- | --- | --- | --- | --- | --- | --- | --- | --- | --- |
|  |  |  |  |  |  |  |  |  |  |  |  |  | Ak^b^ | Km^c^ | Nm^d^ |
| ABH008 | 2012 | H2 | - | + | + | + | + | + | + | + | + | + | R | R | R |
| ABS103 | 2013 | H5 | wound | + | + | + | + | + | + | + | + | + | R | R | R |
| ABS104 | 2013 | H5 | blood | + | + | + | + | + | + | + | + | + | R | R | R |
| ABS178 | 2013 | H5 | wound | + | + | + | + | + | + | + | + | + | R | R | R |
| ABS180 | 2013 | H5 | wound | + | + | + | + | + | + | + | + | + | R | R | R |
| ABS200 | 2013 | H4 | catheter | + | + | + | + | + | + | + | + | + | R | R | R |
| ABS201 | 2013 | H5 | wound | + | + | + | + | + | + | + | + | + | R | R | R |
| ABS226 | 2013 | H5 | - | + | + | + | + | + | + | + | + | + | R | R | R |
| ABS249 | 2013 | H5 | wound | + | + | + | + | + | + | + | + | + | R | R | R |
| ABS288 | 2013 | H5 | wound | + | + | + | + | + | + | + | + | + | R | R | R |
| ABS290 | 2013 | H5 | blood | + | + | + | + | + | + | + | + | + | R | R | R |
| ABM015 | 2011 | H1 | urine | + | + | + | + | + | + | + | + | + | R | R | R |
| ABI031 | 2012 | H3 | fistula | + | + | + | + | + | + | + | + | + | R | R | R |
| ABS094 | 2013 | H5 | wound | + | + | + | + | + | + | + | + | + | R | R | R |
| ABI032 | 2012 | H3 | wound | + | + | + | + | + | + | + | +^a^ | + | R | R | R |
| ABS224 | 2013 | H5 | wound | + | + | + | + | - | + | + | +^a^ | + | R | R | R |
| ABS078 | 2013 | H5 | wound | + | + | + | - | - | + | + | +^a^ | + | R | R | R |
| ABS084 | 2013 | H5 | wound | + | + | + | - | - | + | + | +^a^ | + | R | R | R |
| ABS101 | 2013 | H5 | wound | + | + | + | - | - | + | + | +^a^ | + | R | R | R |
| ABS115 | 2013 | H5 | wound | + | + | + | - | - | + | + | +^a^ | + | R | R | R |
| ABS138 | 2013 | H5 | wound | + | + | + | - | - | + | + | +^a^ | + | R | R | R |
| ABS267 | 2013 | H5 | blood | + | + | + | - | - | + | + | +^a^ | + | R | R | R |
| ABS105 | 2013 | H5 | wound | + | + | + | - | - | - | - | - | - | R | R | R |
| ABS122 | 2013 | H5 | wound | + | + | + | - | - | - | - | - | - | R | R | R |
| ABS258 | 2013 | H5 | wound | + | - | - | - | - | - | - | - | - | R | R | R |
| ABS278 | 2013 | H5 | wound | + | + | + | - | - | - | - | - | - | R | R | R |
| ABS029 | 2012 | H5 | wound | + | + | + | - | - | - | - | - | - | R | R | R |
| ABS035 | 2012 | H5 | wound | + | + | + | - | - | - | - | - | - | R | R | R |
| ABS042 | 2012 | H5 | wound | + | + | + | - | - | - | - | - | - | R | R | R |
| ABS046 | 2012 | H5 | wound | + | + | + | - | - | - | - | - | - | R | R | R |
| ABS081 | 2013 | H5 | wound | - | - | - | - | - | - | - | - | - | I^e^ | R | R |
| ABS085 | 2013 | H5 | wound | + | + | + | - | - | - | - | - | - | R | R | R |
| ABS086 | 2013 | H5 | wound | + | + | + | - | - | - | - | - | - | I^e^ | R | R |
| ABS087 | 2013 | H5 | wound | + | + | + | - | - | - | - | - | - | R | R | R |
| ABS124 | 2013 | H5 | wound | + | + | + | - | - | - | - | - | - | R | R | R |
| ABS155 | 2013 | H5 | wound | + | + | + | - | - | - | - | - | - | R | R | R |
| ABS230 | 2013 | H5 | wound | + | + | + | - | - | - | - | - | - | I^e^ | R | R |
| ABS237 | 2013 | H5 | wound | + | + | + | - | - | - | - | - | - | R | R | R |
| ABS260 | 2013 | H5 | wound | + | + | + | - | - | - | - | - | - | R | R | R |
| ABS263 | 2013 | H5 | wound | + | + | + | - | - | - | - | - | - | R | R | R |
| ABS274 | 2013 | H5 | wound | + | + | + | - | - | - | - | - | - | R | R | R |
| ABS283 | 2013 | H5 | wound | + | + | + | - | - | - | - | - | - | R | R | R |
| ABS219 | 2013 | H5 | wound | + | + | + | - | - | - | - | - | - | R | R | R |
| ABS063 | 2012 | H5 | wound | + | + | + | - | - | - | - | - | - | R | R | R |
| ABS083 | 2013 | H5 | wound | + | + | + | - | - | - | - | - | - | R | R | R |
| ABS121 | 2013 | H5 | wound | + | + | + | - | - | - | - | - | - | R | R | R |
| ABS256 | 2013 | H5 | wound | + | + | + | - | - | - | - | - | - | R | R | R |
| ABS045 | 2012 | H5 | wound | + | + | + | - | - | - | - | - | - | R | R | R |
| ABS062 | 2012 | H5 | wound | + | + | + | - | - | - | - | - | - | R | R | R |
| ABS285 | 2013 | H5 | wound | + | + | + | - | - | - | - | - | - | R | R | R |
| ABS294 | 2013 | H5 | wound | + | + | + | - | - | - | - | - | - | R | R | R |
| ABS206 | 2013 | H5 | wound | + | + | + | - | - | - | - | - | - | R | R | R |
| ABS216 | 2013 | H5 | wound | + | + | + | - | - | - | - | - | - | R | R | R |
| ABS064 | 2012 | H5 | wound | + | - | - | - | - | - | - | - | - | R | R | R |
| ABS186 | 2013 | H5 | blood | + | + | + | - | - | - | - | - | - | R | R | R |

**^a^** The amplicon is larger than the expected size.

^b^ Ak, Amikacin.

^c^ Km, Kanamycin.

^d^ Nm, Neomycin.

^e^ For these isolates, the minimum inhibitory concentration (MIC) of amikacin was determined and its value was 32 μg/mL. The MIC values were categorized as susceptible (S), intermediate (I), or resistant (R) according to the Clinical & Laboratory Standards Institute (CLSI) guidelines.

**Antimicrobial susceptibility testing**

Antimicrobial susceptibility of *A. baumannii* isolates was determined by disk diffusion method. The bacterial suspension (equal to 0.5 McFarland) of isolates was plated on Mueller-Hinton agar (MHA) plates. The disks (Mast Diagnostics Ltd, Bootle, Merseyside, UK) of antibiotics including amikacin (30 μg), kanamycin (30 μg), and neomycin (30 μg) were placed on the plates and incubated at 35± 2°C for 20-24 hours. The diameter of the inhibition zone was measured in millimeters, and the results were interpreted according to the guidelines of the Clinical and Laboratory Standards Institute (CLSI) and the Calibrated Dichotomous Sensitivity (CDS).

**Determination of MIC by broth dilution method**

Minimum inhibitory concentration (MIC) for amikacin was performed by microbroth dilution method using Cation-Adjusted Mueller-Hinton Broth (CAMHB). An initial bacterial suspension was prepared by suspending 3-5 single colonies in 0.9% saline to obtain a turbidity equivalent to the 0.5 McFarland standard. The final 1:100 dilution of the suspension (equal to 5 × 10^5^ CFU/mL) was diluted, and then 100 μl of this suspension was incubated in each well of microplates. Amikacin powder (Sigma Aldrich, St Louis, Missouri, USA) was diluted two-fold ranging from 0.5 to 256 μg/ml. Following incubation at 35± 2°C, the test results were evaluated after 20-24 hours. The MIC values for amikacin were categorized as susceptible (≤16 μg/ml), intermediate (32 μg/ml), or resistant (≥64 μg/ml) according to the Clinical & Laboratory Standards Institute (CLSI) guidelines.
